# Using artificial intelligence to improve governance and public services in Africa

**DOI:** 10.3389/fdata.2026.1835663

**Published:** 2026-06-15

**Authors:** David Mhlanga

**Affiliations:** Department of Financial Governance, University of South Africa, Pretoria, South Africa

**Keywords:** Africa, artificial intelligence, governance, public services, sustainability

## Abstract

African governments continue to face persistent challenges in delivering efficient, transparent, and inclusive public services due to institutional constraints, rapid population growth, and fragmented administrative systems. At the same time, accelerating digital transformation and expanding data ecosystems create new opportunities for governance reform. This study examines the role of Artificial Intelligence (AI) in enhancing public sector performance across welfare targeting, healthcare delivery, tax administration, and urban governance. Drawing on a structured narrative literature review, the paper develops a conceptual framework that conceptualizes governance outcomes as a function of data availability, AI capability, institutional capacity, and human oversight. The findings suggest that AI can improve service delivery by enabling predictive decision-making, reducing administrative inefficiencies, and enhancing targeting accuracy. However, these benefits depend on the alignment between technological adoption and institutional readiness, as weak governance systems may amplify risks such as bias, exclusion, and accountability gaps. The study concludes that AI must be embedded in inclusive, context-sensitive governance strategies to support sustainable development outcomes in Africa.

## 1 Introduction

Governance systems across many African countries continue to operate within complex structural constraints, shaped by limited fiscal capacity, demographic expansion, institutional fragmentation, and rising societal expectations for public service delivery (Azfar et al., 2018; Omweri, 2024). Rapid population growth, projected by the United Nations to double Africa's population by 2050, has intensified pressure on already stretched administrative systems responsible for welfare provision, healthcare delivery, taxation, and urban management (United Nations, 2022; Omweri, 2024). At the same time, public sector institutions frequently rely on legacy administrative structures characterized by fragmented information systems and weak inter-agency coordination, conditions that the African Development Bank identifies as major contributors to inefficiency in service delivery across the continent (African Development Bank, 2023; Mhlanga, 2026). Manual bureaucratic procedures remain prevalent in many government departments, limiting responsiveness and reinforcing governance bottlenecks that undermine citizen trust in state institutions (United Nations, 2022; Omweri, 2024; World Bank, 2025). These institutional limitations directly affect the effectiveness of social protection programmes, revenue mobilization efforts, and urban planning outcomes, particularly in rapidly urbanizing economies such as Nigeria, Kenya, and South Africa (United Nations, 2022; African Development Bank, 2023; Omweri, 2024).

Against this backdrop, Artificial Intelligence (AI) has emerged globally as a transformative technological capability reshaping how governments design and implement public policy (OECD, 2023; Mhlanga, 2025a,b). Advances in machine learning, predictive analytics, and large-scale data processing increasingly enable governments to move from reactive administration toward anticipatory governance models capable of forecasting risks and allocating resources more efficiently (Wirtz and Weyerer, 2023; Mhlanga, 2025a). Countries across Europe and Asia have begun integrating AI into taxation systems, healthcare planning, and welfare administration to enhance efficiency and reduce administrative costs (Saragih et al., 2023; Berryhill et al., 2019). For African states, where institutional capacity constraints often coexist with rapid digital adoption through mobile technologies, AI presents an opportunity to bypass intermediate stages of bureaucratic development, a process frequently described as technological leapfrogging (Foster et al., 2018; Saragih et al., 2023). One important aspect is that digital identification systems and mobile financial platforms already operating across several African economies provide foundational datasets that can support AI-enabled governance innovations (Foster et al., 2018; Saragih et al., 2023; OECD, 2024).

However, technological adoption alone does not automatically translate into improved governance outcomes. Scholars increasingly caution that AI deployment within public administration must be aligned with developmental priorities and institutional realities rather than driven by technological determinism (Foster et al., 2018; Saragih et al., 2023; UNDP, 2024; Mhlanga, 2026). Algorithmic decision-making systems may unintentionally reproduce existing inequalities when datasets exclude informal populations or marginalized communities, a concern particularly relevant in African economies characterized by large informal sectors Foster et al., 2018; Saragih et al., 2023; Eubanks, 2018; World Bank, 2023b). Consequently, AI-driven governance should be understood not as a substitute for public institutions but as a complementary tool that can strengthen state capacity, enhance transparency, and improve policy targeting when supported by appropriate regulatory oversight and human accountability mechanisms (Mhlanga, 2025b, 2026). The central governance challenge facing African states, therefore, lies in harnessing Artificial Intelligence to expand inclusion and public value while avoiding new forms of digital exclusion or technological dependency (Mhlanga, 2025b, 2026). African countries exhibit substantial diversity in governance systems, digital infrastructure, and institutional capacity. Therefore, AI adoption and its impact on public service delivery should be understood within the context of specific national conditions rather than as a uniform continental experience. To provide context for the governance challenges facing African public institutions and the potential role of Artificial Intelligence interventions, Table 1 summarizes key administrative constraints alongside corresponding AI-enabled solutions.

**Table 1:** Governance challenges and AI intervention areas in Africa.

Governance challenge	Traditional limitation	AI-based solution	Expected governance outcome
Welfare targeting	Manual beneficiary identification	Predictive poverty analytics	Reduced exclusion errors
Healthcare delivery	Fragmented data systems	AI diagnostics and forecasting	Improved service access
Tax administration	Low compliance	AI risk profiling	Increased revenue mobilization
Urban governance	Reactive planning	Smart city analytics	Efficient service delivery

Source: Authors' Analysis.

Table 1 provides the governance challenges and AI intervention areas in Africa. It summarizes the major governance constraints affecting public service delivery across African countries and maps these challenges to corresponding Artificial Intelligence (AI) intervention areas. The table illustrates how AI technologies can address longstanding administrative inefficiencies by enabling predictive analytics, automation, and data-driven decision-making. The table provides a conceptual foundation for understanding AI as an enabling instrument for developmental governance rather than merely a technological upgrade, through linking governance problems with technological solutions and expected outcomes.

## 2 Methodology

### 2.1 Research design

This study adopts a qualitative research design grounded in conceptual analysis and a policy-oriented literature review to examine how Artificial Intelligence (AI) can improve governance and public service delivery across African countries (Kitchin, 2019; Sun and Medaglia, 2019; Wirtz et al., 2019). Qualitative approaches are widely used in governance and digital transformation research to explore emerging technological phenomena and institutional developments where empirical data remain limited (Sun and Medaglia, 2019; Wirtz et al., 2019). The research synthesizes insights from peer-reviewed academic literature and scholarly publications to analyse emerging AI applications in welfare targeting, healthcare systems, tax administration, and urban governance (Berryhill et al., 2019; Wirtz et al., 2019). Literature synthesis enables the study to consolidate knowledge from multiple studies and develop a conceptual understanding of how technological innovations influence public-sector governance systems (Okoli and Schabram, 2015; Kitchin, 2019). The study follows a desk-based analytical methodology, which is commonly applied in policy and governance studies that examine institutional frameworks, technological developments, and regulatory systems rather than generating primary field data (Kitchin, 2019; Okoli and Schabram, 2015). Desk-based research allows scholars to integrate evidence from diverse disciplinary perspectives and analyse complex governance challenges associated with emerging technologies (Sun and Medaglia, 2019). This approach facilitates the integration of insights from public administration, development economics, digital governance, and artificial intelligence, enabling a comprehensive assessment of how AI-enabled systems may enhance state capacity, administrative efficiency, and data-driven decision-making within public sector institutions (Wirtz et al., 2019; Russell and Norvig, 2021). Such conceptual approaches are widely used to examine how digital technologies reshape governance systems and public policy implementation in both developed and developing economies (Berryhill et al., 2019).

### 2.2 Literature review strategy

This study adopts a structured narrative literature review approach to examine the role of Artificial Intelligence (AI) in governance and public service delivery across African countries. Academic databases, including Scopus, Web of Science, and Google Scholar, were systematically searched to identify relevant literature. The search strategy employed keywords such as “Artificial Intelligence governance Africa,” “digital government AI,” “AI public sector innovation,” and “predictive governance systems.” The review focused on literature published between 2015 and 2025 to capture recent developments in AI and digital governance. Inclusion criteria required sources to be peer-reviewed journal articles, books, or institutional reports (e.g., World Bank, OECD, UNDP) with direct relevance to governance, public service delivery, or digital transformation. Studies focusing solely on technical AI development without governance implications were excluded. The collected literature was analyzed using a thematic approach, allowing the identification of recurring patterns across governance sectors, including welfare targeting, healthcare systems, tax administration, and urban governance. This approach supports the development of a conceptual understanding of AI-enabled governance as a socio-technical system. A total of approximately 85–110 sources were initially identified through database searches (Scopus, Web of Science, Google Scholar). After removing duplicates and screening titles and abstracts for relevance, 52 studies were retained for full-text review. Of these, 38 sources were included in the final analysis based on relevance to AI governance, public service delivery, and African contexts. The selection process followed three stages: initial identification using keyword searches, Screening based on relevance to governance and AI and Eligibility assessment based on conceptual and policy contribution. The selected studies were synthesized using thematic analysis, grouping findings into four governance domains: welfare, healthcare, taxation, and urban governance.

### 2.3 Sources of data

The analysis is based on secondary data drawn from a range of institutional and academic sources, including peer-reviewed journal articles, policy reports from international organizations, and digital governance strategies developed by African governments, as summarized in Table 2. These sources were selected based on their relevance, credibility, and contribution to understanding AI-enabled governance in developing country contexts. The use of diverse data sources enables a comprehensive, multidisciplinary assessment of how Artificial Intelligence is shaping public-sector transformation across Africa.

**Table 2:** Data sources used in the study.

Data source category	Type of documents	Examples of sources	Purpose in the study
Academic literature	Peer-reviewed journal articles and books	Public administration, AI governance, and development economics journals	Theoretical foundation of AI governance
International policy reports	Institutional reports and governance assessments	World Bank, OECD, AfDB, UNDP, IMF	Evidence on governance challenges and digital transformation
Government policy documents	National digital strategies and AI policies	Rwanda Digital Strategy, Kenya e-Government systems	Understanding national AI governance frameworks
Technology and governance reports	Digital economy and infrastructure assessments	ITU, UNESCO, UNECA	Evaluation of digital infrastructure readiness

Source: Authors' Analysis

These sources, outlined in Table 2, provide a comprehensive evidence base for examining governance challenges and the potential of AI to improve administrative efficiency and service delivery. These sources were systematically selected based on relevance, credibility, and contribution to understanding AI-enabled governance, ensuring analytical consistency across the reviewed literature.

### 2.4 Analytical framework

The study uses a thematic analytical framework to examine the role of Artificial Intelligence across four core governance sectors. These sectors represent key areas where digital technologies can significantly improve public service delivery. Table 3 outlines the governance sectors analyzed in the study. As shown in Table 3, governance systems across the four sectors are characterized by persistent structural inefficiencies and fragmented administrative processes. In welfare and social protection, inaccurate beneficiary targeting arises from reliance on manual identification methods and outdated datasets. In healthcare systems, limited diagnostic capacity and fragmented health data constrain timely and effective service delivery. Tax administration is similarly affected by weak compliance monitoring, contributing to tax evasion and reduced revenue mobilization. In urban governance, rapid urbanization is met with inefficient and reactive service delivery systems. These challenges persist because traditional governance approaches depend on manual processes, limited data integration, and weak institutional coordination, ultimately reducing the effectiveness of public service delivery.

**Table 3:** Governance sectors analyzed in the study.

Governance sector	Traditional governance challenge	AI application	Expected governance outcome
Welfare and social protection	Inaccurate beneficiary targeting	Machine learning poverty identification	Improved targeting accuracy
Healthcare systems	Limited diagnostic capacity and fragmented health data	AI diagnostics and predictive health analytics	Improved healthcare access
Tax administration	Tax evasion and weak compliance monitoring	AI risk profiling and anomaly detection	Increased revenue mobilization
Urban governance	Rapid urbanization and inefficient service delivery	Smart city analytics and predictive urban planning	Improved urban management

Source: Authors' Analysis.

The framework enables the study to systematically analyse how AI technologies transform governance functions by enabling predictive decision-making, automation, and improved coordination across public-sector institutions. As illustrated in Table 3, the integration of Artificial Intelligence enables a transition toward predictive, data-driven governance across all four sectors. In welfare and social protection, machine learning improves targeting accuracy by analyzing multidimensional datasets to identify vulnerable populations more precisely. In healthcare systems, AI diagnostics and predictive analytics enhance access to services by enabling early detection and more efficient allocation of limited healthcare resources. In tax administration, AI-based risk profiling and anomaly detection increase revenue mobilization by identifying compliance risks and irregular patterns more effectively than traditional methods. In urban governance, smart city analytics and predictive planning improve service delivery by forecasting infrastructure demand and enabling proactive decision-making As reflected in Table 3, AI enhances governance outcomes because it reduces information asymmetry, accelerates decision-making processes, and strengthens coordination across public-sector institutions.

### 2.4 Comparative country analysis

To assess variations in AI governance readiness across Africa, the study includes a comparative analysis of selected African countries that have begun implementing digital governance systems. These countries were selected based on differences in digital infrastructure development, e-government maturity, and national AI policy frameworks. Table 4 outlines the criteria for country selection used in the study.

**Table 4:** Criteria for country selection.

Selection criterion	Explanation
Digital infrastructure development	Availability of broadband connectivity and data systems
National AI strategy	Presence of AI policy frameworks or digital transformation strategies
E-government maturity	Level of digital public service delivery
Institutional capacity	Ability of public institutions to manage AI systems
Data governance frameworks	Existence of data protection and privacy regulations

Source: Authors Analysis.

The comparative approach allows the study to identify patterns of AI adoption across African governance systems and highlight differences in institutional readiness. All these were critical for the development of the conceptual Framework proposed in this study. To address the variation in AI adoption across governance systems, this study adopts a comparative perspective focusing on selected African countries that have demonstrated progress in digital governance initiatives. Rather than treating the continent as a homogeneous entity, the analysis examines differences in digital infrastructure, institutional capacity, and AI application across national contexts. The countries included, Rwanda, Kenya, South Africa, and Ghana, represent varying levels of digital maturity and governance readiness. These cases are selected to illustrate how contextual factors shape the adoption and effectiveness of AI-enabled public service delivery. Table 5 presents a comparative overview of AI governance readiness across these countries, highlighting key dimensions including infrastructure, applications, governance outcomes, and constraints.

**Table 5:** Comparative AI governance readiness in selected African countries.

Country	Digital Infrastructure	AI Application	Governance Outcome	Key Constraint
Rwanda	High (strong e-government platforms)	Digital service delivery (Irembo)	Improved efficiency	Limited scalability
Kenya	Strong mobile ecosystem	Digital ID & mobile governance	Financial inclusion	Data fragmentation
South Africa	Advanced systems	AI in tax compliance	Revenue improvement	Inequality
Ghana	Moderate	Smart governance systems	Improved access	Institutional capacity

Source: Author's Analysis.

Table 5 demonstrates that AI governance readiness across African countries is uneven and shaped by context-specific institutional and technological conditions. Rwanda emerges as a leader in integrated digital governance systems, particularly through platforms such as Irembo, which enhance administrative efficiency. However, scalability challenges remain, especially in extending services to rural populations. Kenya's strong mobile ecosystem supports innovations in digital identification and financial inclusion, yet fragmented data systems continue to limit the full integration of AI-driven governance. South Africa has advanced institutional capacity and has successfully applied AI in areas such as tax administration, thereby improving revenue mobilization. Non-etheless, persistent socio-economic inequalities affect the equitable distribution of these benefits. Ghana represents an emerging case where digital governance systems are expanding, but institutional and technical capacity constraints remain key barriers. The comparative analysis highlights that AI-enabled governance cannot be understood as a uniform process across Africa. Instead, its effectiveness depends on the alignment between digital infrastructure, institutional capacity, and regulatory frameworks. These findings reinforce the argument that successful AI adoption in governance requires context-sensitive strategies that account for national differences in development trajectories and administrative systems. The observed differences across countries can be explained by variations in institutional capacity, digital infrastructure maturity, and governance models. Rwanda's strong centralized governance enables coordinated digital implementation, whereas Kenya's decentralized and market-driven ecosystem promotes innovation but leads to data fragmentation. South Africa's relatively advanced institutional systems support AI deployment in taxation, yet structural inequality limits inclusive outcomes. Ghana's moderate performance reflects transitional institutional development, where policy ambition exceeds implementation capacity.

### 2.5 Conceptual framework

The study proposes a conceptual governance framework that illustrates how Artificial Intelligence can enhance public-sector performance in Africa. First, the study starts with the predictive governance function shown below to conceptualize how Artificial Intelligence contributes to improved public sector governance. The conceptual framework is grounded in socio-technical systems theory, which emphasizes the interaction between technological innovation and institutional structures in shaping governance outcomes (Janowski, 2015; Wirtz et al., 2019). It also draws on digital governance theory, which highlights the transition from traditional bureaucratic systems to data-driven and predictive governance models (OECD, 2023). Furthermore, the framework aligns with the literature on algorithmic governance, which examines how artificial intelligence systems influence public-sector decision-making processes and accountability structures (Kitchin, 2019). These theoretical foundations justify integrating data availability, AI capability, institutional capacity, and human oversight as key dimensions of AI-enabled governance.

This framework advances existing digital governance literature by integrating technological, institutional, and human dimensions into a single functional model. Prior studies on artificial intelligence in the public sector have largely emphasized technological capabilities and application domains, often focusing on efficiency gains, automation, and service optimisation (e.g., Berryhill et al., 2019; Wirtz et al., 2019; Russell and Norvig, 2021). Similarly, digital governance research has primarily examined the transition from traditional bureaucratic systems to data-driven and platform-based governance models, with limited integration of institutional and human oversight dimensions (Janowski, 2015; OECD, 2023). While emerging work on algorithmic governance acknowledges the role of data and analytics in shaping decision-making processes, it often treats governance outcomes as a function of technological systems rather than a socio-technical interaction between data, institutions, and human agency (Kitchin, 2019; Sun and Medaglia, 2019). In contrast, the proposed framework conceptualizes governance outcomes as a product of interaction between data ecosystems, institutional capacity, and human oversight, providing a more comprehensive lens for analyzing AI-enabled governance in developing country contexts where institutional constraints and capacity gaps play a decisive role. This integrative perspective is particularly important in African contexts, where governance outcomes are shaped not only by technological adoption but by the alignment between infrastructure, institutional readiness, and accountability structures.

### 2.6 Predictive governance function

To conceptualize how Artificial Intelligence contributes to improved public sector governance, this study proposes a Predictive Governance Function, expressed as *G* = *f*(*D, A, I, H*) shown in Equation 1 below.


G=f(D,A,I,H) (1)

Where:

G = Governance performance

D = Data availability

A = AI capability

I = Institutional capacity

H **=** Human capital

The function in Equation 1 illustrates that governance performance (*G*) is influenced by the interaction between data ecosystems (*D*), artificial intelligence capabilities (*A*), institutional capacity (*I*), and human oversight **(***H***)**. Rather than viewing AI as an autonomous technological solution, the model emphasizes that effective AI-enabled governance emerges from the combined operation of digital infrastructure, analytical technologies, administrative institutions, and accountable decision-making structures. The first component of the function *(D)* refers to data availability. In contemporary digital governance systems, data availability and quality serve as foundational inputs for AI-driven policy analysis (He et al., 2025; Androutsopoulou et al., 2025). Governments increasingly rely on large-scale administrative datasets, digital identification systems, mobile transaction records, and sensor-based information streams to monitor social and economic activities in real time (Kitchin, 2019; He et al., 2025; Androutsopoulou et al., 2025). These data ecosystems enable public institutions to identify patterns in service demand, poverty dynamics, healthcare utilization, and urban mobility, thereby providing the empirical basis for predictive governance systems. However, data alone does not improve governance outcomes unless supported by analytical technologies that can extract actionable insights.

The second component of the function, AI analytical capability **(***A***)**, refers to machine learning algorithms, predictive analytics, and automated decision-support systems used to interpret large datasets and generate policy-relevant insights. AI technologies enable governments to move beyond reactive administrative models toward anticipatory governance, in which risks and service needs can be forecast before crises occur (Wirtz et al., 2019; Pashentsev and Kolotaev, 2025; He et al., 2025). For example, predictive analytics can estimate future healthcare demand, identify potential tax compliance risks, or forecast infrastructure pressures in rapidly urbanizing cities. Such analytical capabilities significantly enhance public institutions' ability to allocate resources efficiently and design targeted policy interventions. Nevertheless, the effectiveness of AI-enabled governance also depends critically on institutional capacity (*I*). Institutional capacity includes the administrative structures, regulatory frameworks, technical expertise, and organizational coordination required to manage digital governance systems. Scholars of digital government emphasize that technological innovation alone cannot transform governance unless institutions possess the capacity to interpret analytical outputs, integrate them into policy processes, and ensure regulatory oversight (Janowski, 2015; Sun and Medaglia, 2019; Erkut, 2020). Weak institutional environments may limit the benefits of AI adoption, leading to fragmented digital initiatives rather than systemic governance transformation.

The final component of the function, human oversight (*H*), underscores the need to maintain democratic accountability and ethical governance within algorithmic decision-making systems. AI technologies can assist public officials in analyzing complex datasets and generating policy recommendations, but final decision-making authority must remain subject to human judgment and public accountability mechanisms (Berryhill et al., 2019; Mhlanga, 2023, 2026). Human oversight ensures that algorithmic decisions remain transparent, contestable, and aligned with broader public interest objectives. Without such safeguards, automated governance systems risk reinforcing biases embedded in historical datasets or reducing transparency in administrative decision-making (Berryhill et al., 2019; Mhlanga, 2023, 2026). Taken together, the Predictive Governance Function highlights that AI-enabled governance should be understood as a socio-technical system rather than a purely technological intervention. Effective governance outcomes arise from the interaction between digital data infrastructures, advanced analytical capabilities, institutional readiness, and accountable human decision-making. In the context of African public administration, this framework underscores the importance of strengthening digital infrastructure, developing AI expertise within public institutions, and establishing robust governance frameworks to oversee algorithmic decision-making systems. By integrating these elements, African governments can transition toward predictive governance models that improve policy responsiveness, enhance service delivery efficiency, and support inclusive development outcomes. This study conceptualizes AI-enabled governance through a predictive governance function that illustrates the interaction between digital data ecosystems, artificial intelligence capability, institutional capacity, and human oversight. The study proposes a conceptual governance framework that illustrates how Artificial Intelligence can enhance public-sector performance in Africa.

**Figure 1:**
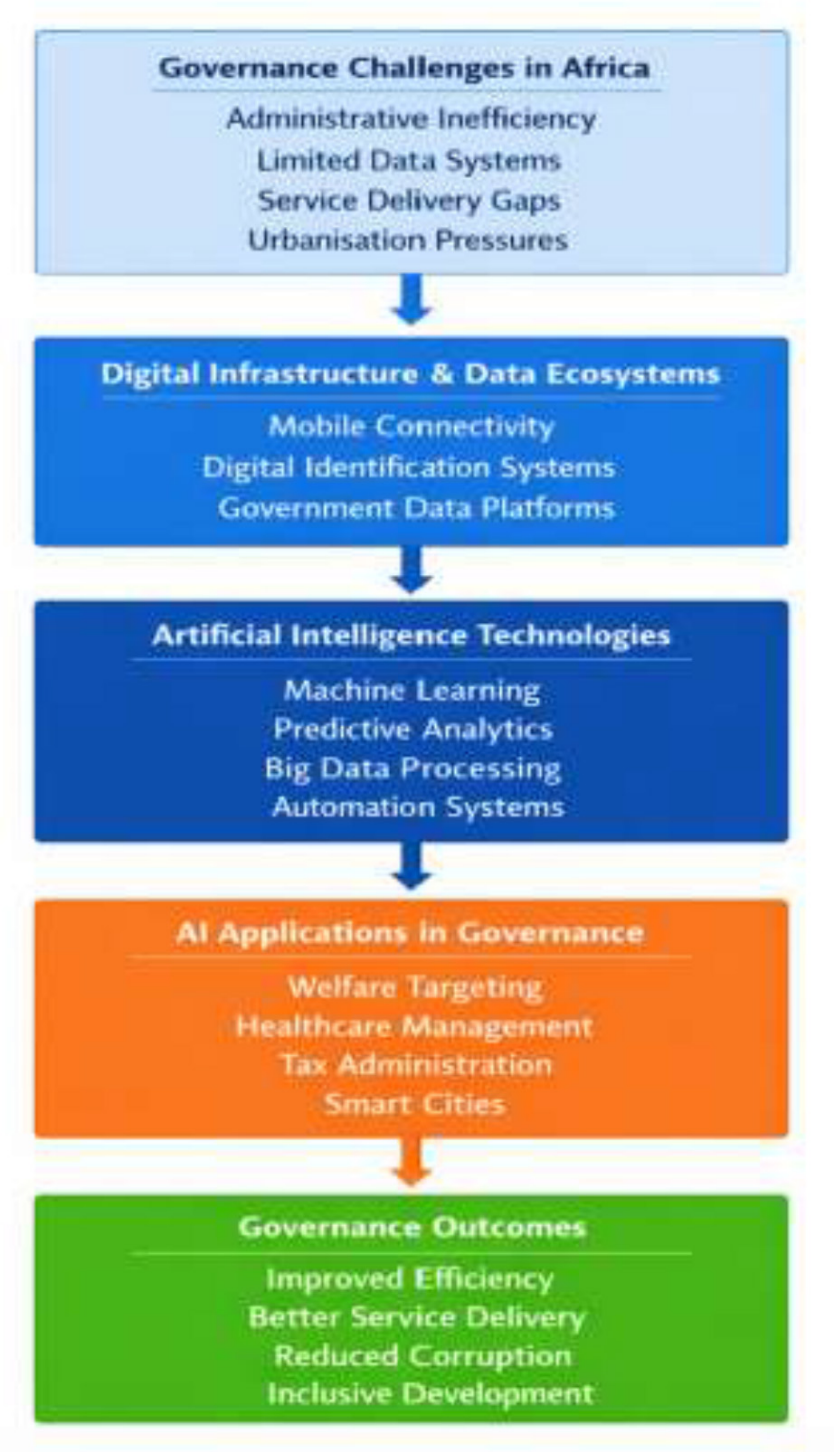
Conceptual framework: AI-enabled governance transformation. Source: Authors' analysis.

This conceptual framework in Figure 1 illustrates how digital infrastructure and AI technologies interact to strengthen governance systems, enabling governments to transition from reactive to predictive, data-driven governance.

### 2.7 Artificial intelligence and digital governance

Artificial Intelligence (AI) broadly refers to computational systems capable of processing large volumes of structured and unstructured data, identifying patterns, learning iteratively from experience, and supporting automated or semi-automated decision-making processes (Russell and Norvig, 2021; Mhlanga, 2023). Contemporary governance applications increasingly rely on machine learning and predictive analytics to enhance administrative decision-making beyond traditional rule-based bureaucratic systems (Russell and Norvig, 2021; Mhlanga, 2023). Within public sector contexts, AI enables governments to transition from reactive governance, where policy responses follow crises, to predictive governance models that anticipate risks, forecast service needs, and support evidence-based intervention planning (Russell and Norvig, 2021; OECD, 2023; Wirtz and Weyerer, 2023). This shift represents a significant transformation in public administration, aligning governance practices with real-time data environments characteristic of digitally connected societies.

Digital governance supported by AI enables public institutions to integrate previously fragmented administrative datasets across ministries and agencies, thereby improving coordination and policy coherence (Sun and Medaglia, 2019; World Bank, 2025). Through advanced analytics, governments can analyse citizen behavior patterns, monitor service utilization, and forecast demand for healthcare, education, and social protection programmes (Sun and Medaglia, 2019; World Bank, 2025). Machine learning algorithms are increasingly capable of detecting anomalies within administrative systems, including fraud risks, revenue leaks, and inefficiencies in procurement processes, while simultaneously identifying vulnerable populations that require targeted policy interventions (Sun and Medaglia, 2019; World Bank, 2025). Such capabilities enhance operational efficiency and allow governments to allocate scarce public resources more strategically, an especially critical consideration for developing economies facing fiscal constraints (Sun and Medaglia, 2019; World Bank, 2025).

Across Africa, the ongoing digital transformation is laying the foundation for AI-enabled governance (Alice and Ebuka, 2024; Nnaji et al., 2026). The rapid expansion of mobile connectivity, now exceeding 80 per cent population coverage in Sub-Saharan Africa, according to the International Telecommunication Union (International Telecommunication Union, 2023), has generated large-scale digital datasets through mobile money platforms, digital identity programmes, and electronic government services. Countries such as Rwanda have implemented national digital governance frameworks integrating online public services through platforms like Irembo, while Kenya's digital identification and mobile payment ecosystem supports data-driven public administration initiatives (Kemp, 2024; Alice and Ebuka, 2024; Nnaji et al., 2026). Similarly, South Africa and Ghana have expanded e-government systems to improve service accessibility and administrative transparency, reflecting a broader continental shift toward digitally mediated governance structures (UNECA, 2023; Alice and Ebuka, 2024; Nnaji et al., 2026). These developments demonstrate that digital infrastructure investments are increasingly serving as precursors to AI adoption in public administration.

Despite these advancements, AI adoption across African governance systems remains uneven and constrained by structural challenges (Mhlanga, 2026; Nnaji et al., 2026). Persistent infrastructure deficits, including unreliable electricity supply and limited broadband penetration in rural regions, continue to restrict large-scale deployment of data-intensive technologies (UNESCO, 2021; African Union, 2020). Equally significant is the shortage of specialized technical expertise within public institutions, which limits governments' capacity to design, interpret, and regulate algorithmic systems effectively (African Union, 2020; UNESCO, 2021). Regulatory uncertainty surrounding data protection, algorithmic accountability, and cross-border data governance further complicates implementation efforts (Mhlanga, 2026; Nnaji et al., 2026). Consequently, successful integration of Artificial Intelligence into African governance systems requires not only technological innovation but also institutional readiness, regulatory development, and sustained investment in human capital capable of managing digital transformation responsibly (Mhlanga, 2026; Nnaji et al., 2026). Table 6 presents major applications of Artificial Intelligence across core public-sector governance functions and illustrates how digital technologies are transforming administrative decision-making processes.

**Table 6:** AI applications in public sector governance.

Governance function	AI technology used	Application example	African case
Public administration	Machine learning	Resource allocation	Rwanda
Fraud detection	Predictive analytics	Procurement monitoring	South Africa
Citizen services	Chatbots	Digital government portals	Kenya
Policy planning	Big data analytics	Demand forecasting	Ghana

Source: Authors' Analysis.

Table 6 presents key applications of Artificial Intelligence across core public-sector governance functions. It demonstrates how machine learning, predictive analytics, and automated service platforms are increasingly integrated into administrative systems to enhance coordination, transparency, and policy responsiveness. The inclusion of African country examples highlights emerging regional adoption patterns and illustrates the practical transition from traditional bureaucratic governance toward digitally enabled public administration.

### 2.8 Artificial intelligence in welfare targeting and social protection

Social protection systems across Africa play a critical role in addressing poverty, vulnerability, and inequality, yet many programmes continue to face persistent challenges related to beneficiary identification, targeting accuracy, and administrative efficiency (Abay et al., 2021, 2023). Traditional welfare delivery mechanisms often rely on outdated household surveys or manual verification procedures that struggle to capture rapidly changing socio-economic conditions, particularly within informal economies that employ most of Africa's labor force (World Bank, 2022). As (Devereux and Sabates-Wheeler 2015) observe, weak data systems frequently result in both inclusion errors, in which benefits reach unintended recipients, and exclusion errors that affect the most vulnerable populations in need of assistance. These limitations undermine the effectiveness of social protection programmes and reduce public confidence in government redistribution mechanisms (World Bank, 2025; Mhlanga, 2023, 2026).

Artificial Intelligence offers new possibilities for improving welfare targeting through data-driven identification of socio-economic vulnerability (Jean et al., 2016; UNDP, 2024). Machine learning systems can analyse multidimensional datasets, including demographic indicators, consumption behavior, satellite imagery, and mobile transaction records, to generate dynamic poverty assessments that evolve in real time rather than relying solely on periodic surveys (Jean et al., 2016; UNDP, 2024). Increasingly, governments are exploring predictive analytics to anticipate livelihood shocks from climate events, food insecurity, or economic disruptions, thereby enabling preventive rather than reactive welfare interventions (Jean et al., 2016; UNDP, 2024). Such approaches represent a shift toward adaptive social protection systems that can respond more effectively to complex development risks across African economies (Jean et al., 2016; UNDP, 2024).

Several African countries have begun integrating digital technologies that provide foundational infrastructure for AI-supported welfare governance. Digital identification programmes, such as Nigeria's National Identity Number system and Kenya's Huduma digital service platforms, enable governments to link citizens to social benefits through verified databases, reducing duplication and administrative leakages (Dzreke et al., 2025; Trikanad and Bhandari, 2022). Mobile money ecosystems further enhance welfare delivery by facilitating direct benefit transfers, improving transparency, and lowering transaction costs associated with cash-based distribution systems (Gelb and Metz, 2018; Trikanad and Bhandari, 2022; Suri et al., 2021). During the COVID-19 pandemic, countries including Togo deployed digitally enabled emergency cash transfer programmes that used algorithmic targeting to identify informal workers affected by lockdown measures, demonstrating the practical potential of data-driven welfare systems in crisis contexts (Aiken et al., 2022; Dzreke et al., 2025; Trikanad and Bhandari, 2022).

Despite these advances, integrating AI into welfare governance raises significant ethical and institutional concerns. Algorithmic decision-making systems may inadvertently reproduce structural inequalities where datasets fail to adequately represent rural populations, women, or informal workers who remain digitally invisible (Eubanks, 2018; Dzreke et al., 2025; Trikanad and Bhandari, 2022). Scholars caution that automated welfare systems risk transforming social protection into systems of surveillance if adequate safeguards for privacy and accountability are absent (Eubanks, 2018; Taylor, 2023). Furthermore, excessive reliance on externally developed AI technologies may reduce domestic policy autonomy, particularly where governments lack the technical capacity to audit or interpret algorithmic outcomes (Eubanks, 2018; Taylor, 2023). Consequently, effective AI-enabled welfare systems must retain human oversight mechanisms and transparent appeal processes to ensure fairness and legitimacy in public decision-making (Dzreke et al., 2025; Trikanad and Bhandari, 2022). Ultimately, Artificial Intelligence should be viewed as an enabling tool rather than a replacement for social policy institutions because when combined with strong governance frameworks, inclusive data strategies, and institutional accountability, AI has the potential to enhance targeting efficiency, expand coverage, and improve responsiveness within African social protection systems (Dzreke et al., 2025; Trikanad and Bhandari, 2022). The central policy challenge is to ensure that technological innovation strengthens social inclusion rather than reinforcing existing patterns of marginalization. To demonstrate the role of Artificial Intelligence in strengthening social protection delivery, Table 7 outlines key AI-supported approaches to welfare targeting and beneficiary identification.

**Table 7:** AI in welfare targeting systems.

Welfare function	Data source	AI method	Policy benefit
Poverty identification	Mobile data	ML classification	Accurate targeting
Cash transfers	Digital ID	Algorithmic matching	Reduced leakage
Shock response	Climate data	Predictive modeling	Early intervention
Informal worker support	Transaction data	Pattern recognition	Inclusion expansion

Source: Authors' Analysis.

Table 7 shows the AI in welfare targeting systems. This table outlines how Artificial Intelligence technologies improve welfare targeting and social protection delivery by integrating multidimensional datasets. It highlights the role of machine learning models in reducing inclusion and exclusion errors commonly associated with manual beneficiary identification systems. The table further demonstrates how AI-supported welfare governance enables adaptive social protection mechanisms that respond dynamically to poverty shocks, climate risks, and economic disruptions. Table 8 provides a structured synthesis of the key scholarly and policy literature underpinning this study, organized across five core governance themes. The table highlights how different strands of research collectively inform the conceptualization of AI-enabled governance in Africa, linking foundational governance challenges with emerging technological, institutional, and ethical considerations.

**Table 8:** Summary of references by governance theme.

Theme	Key references	Role in the paper
Governance challenges	Azfar et al. (2018); Omweri (2024); African Development Bank (2023); United Nations (2022); World Bank (2023a)	Establish the baseline of institutional fragmentation, rapid population growth, and manual bureaucratic bottlenecks in Africa.
AI & predictive governance	OECD (2023); Mhlanga (2025a); Berryhill et al. (2019); Russell and Norvig (2021); He et al. (2025)	Define AI as a tool for shifting from reactive administration to anticipatory, data-driven governance models.
Welfare & social protection	Jean et al. (2016); UNDP (2024); Dzreke et al. (2025); Aiken et al. (2022); Abay et al. (2021)	Demonstrate how machine learning and digital IDs improve beneficiary targeting and reduce exclusion errors in aid delivery.
Institutional & ethical risks	Eubanks (2018); Taylor (2023); Mhlanga (2026); Foster et al. (2018); UNESCO (2021)	Highlight risks of algorithmic bias, digital exclusion, surveillance, and the need for robust human oversight.
Digital infrastructure	International Telecommunication Union (2023); African Union (2020); Kemp (2024); UNECA (2023)	Provide evidence on the expansion of mobile connectivity and national digital strategies (e.g., Rwanda, Kenya) as foundations for AI.
Methodological framework	Kitchin (2019); Sun and Medaglia (2019); Wirtz et al. (2019); Okoli and Schabram (2015)	Support the qualitative research design and the conceptual Predictive Governance Function.

Source: Authors' Analysis.

Table 8 demonstrates that the literature on AI-enabled governance in Africa is both multidisciplinary and layered, spanning governance theory, technological innovation, social policy, and institutional analysis. The first theme, governance challenges, establishes the structural constraints facing many African states, including institutional fragmentation, demographic pressures, and inefficiencies associated with manual administrative systems. These foundational issues provide the context for considering AI interventions. The second theme, AI and predictive governance, shifts the discussion toward the transformative potential of artificial intelligence. The cited works collectively frame AI as an enabler of anticipatory governance, enabling governments to move from reactive service delivery to proactive, data-driven decision-making. In the welfare and social protection literature, empirical evidence shows that digital technologies, particularly machine learning and digital identification systems, can improve targeting, reduce leakage, and minimize exclusion errors in public service delivery. The fourth theme, institutional and ethical risks, introduces a critical perspective by highlighting the unintended consequences of AI adoption. These include algorithmic bias, digital exclusion, surveillance concerns, and the need for accountability mechanisms and human oversight to ensure equitable outcomes.

Finally, the themes of digital infrastructure and methodological framework underscore the enabling conditions for AI adoption. While infrastructure-related studies point to expanding connectivity and national digital strategies as foundational pillars, the methodological literature supports the study's qualitative and conceptual approach, particularly the development of the Predictive Governance Function. Taken together, the table illustrates that AI-enabled governance is not merely a technological transition but a complex socio-technical transformation that requires alignment among digital capabilities, institutional systems, and ethical safeguards.

The literature reviewed in Table 8 collectively suggests that, while artificial intelligence presents a significant opportunity for technological leapfrogging in African governance systems, its effectiveness is not determined solely by technology (African Development Bank, 2023; OECD, 2023). Rather, governance outcomes emerge from a socio-technical interplay of multiple factors (Kitchin, 2019; Wirtz et al., 2019). These include the availability of high-quality, real-time data, which underpins effective predictive analytics (United Nations, 2022; World Bank, 2023a), as well as the deployment of advanced analytical capabilities such as machine learning to support anticipatory decision-making (Russell and Norvig, 2021; Berryhill et al., 2019). Equally important is the strength of institutional capacity and regulatory frameworks, which determine the extent to which AI systems can be effectively governed and integrated into public administration (UNESCO, 2021; Taylor, 2023). Furthermore, the presence of skilled human capital and robust ethical oversight mechanisms is critical to mitigating risks such as algorithmic bias, digital exclusion, and surveillance concerns (Eubanks, 2018; Foster et al., 2018; Mhlanga, 2026). Consequently, the success of AI-enabled governance in Africa depends on the extent to which these elements are developed in a coordinated and context-sensitive manner (African Union, 2020; UNECA, 2023).

## 3 Conclusion

This study has examined the role of AI in transforming governance and public service delivery across African countries, highlighting its potential to shift public administration from reactive, bureaucratic processes to predictive, data-driven decision-making systems. Across key sectors including welfare, healthcare, taxation, and urban governance, AI demonstrates clear potential to enhance efficiency and targeting by enabling real-time data analysis, anticipatory policy responses, and more effective allocation of scarce public resources. However, the findings underscore that technological adoption alone does not guarantee improved governance outcomes. The effectiveness of AI-enabled systems depends on the alignment between data availability, institutional capacity, human capital, and regulatory frameworks. In this study, these elements are conceptualized as an interconnected socio-technical system in which governance performance emerges from the interaction between technological capability and institutional readiness. This perspective extends existing literature by moving beyond technology-centric explanations and emphasizing the structural conditions under which AI can generate public value in developing country contexts. The analysis also highlights critical risks associated with AI deployment. In contexts characterized by weak institutions and uneven data ecosystems, AI systems may reinforce existing inequalities, as incomplete or biased datasets can lead to exclusionary outcomes and reduced accountability in decision-making. This reinforces the need for robust governance frameworks that ensure transparency, ethical oversight, and human accountability. From a policy perspective, governments should prioritize coordinated investments in digital infrastructure, data governance systems, and local technical capacity. Importantly, AI strategies must be context-sensitive, recognizing the diversity of institutional environments across African countries rather than assuming uniform adoption pathways. This study is limited by its reliance on secondary data and conceptual analysis. Future research should incorporate empirical and case-based approaches to test the proposed framework and evaluate the real-world impacts of AI adoption on governance performance across different institutional settings.
